# The microbiota-host metabolic axis in cervical cancer: from homeostatic disruption to mechanisms of therapy resistance

**DOI:** 10.3389/fcimb.2026.1890330

**Published:** 2026-08-05

**Authors:** Yulu He, Yuan Zhao, Shang Ju, Peiwen Zhou, Junzhuo Si, Yijia Pan, Yanfang Jiang

**Affiliations:** Genetic Diagnosis Center, The First Hospital of Jilin University, Changchun, China

**Keywords:** cervical cancer, HPV - human papillomavirus, metabolic reprogramming, therapy resistance, vaginal microbiota

## Abstract

Persistent infection with high-risk human papillomavirus (HR-HPV) is a prerequisite for cervical carcinogenesis. In this process, the vaginal microbiota, as a key component of the cervical microenvironment, plays a significant role in facilitating viral persistence and malignant transformation. Moving beyond the conventional focus on compositional shifts, this review systematically examines the dynamic remodeling of the host-microbiota co-metabolic network during the progression from health to invasive cervical cancer. It explores in depth the interactive mechanisms through which metabolites derived from a dysbiotic microbiota exacerbate epithelial barrier damage, modulate local immune evasion, and activate oncogenic signaling pathways, with particular emphasis on how co-metabolic imbalance drives therapeutic resistance. By integrating these insights, this review aims to advance the mechanistic understanding of microbial contributions to cervical cancer development and to inform novel strategies for overcoming treatment resistance through microbiota-targeted interventions.

## 1 Introduction

Cervical cancer is the fourth most common cancer among women worldwide, with approximately 660,000 new cases and 348,000 deaths in 2022, posing a significant global public health threat (Bray et al., 2024). While persistent infection with HR-HPV is established as the central etiologic factor, clinical observations show that 80%–90% of infected individuals can clear the virus through their own immunity, with only a small minority progressing to malignancy (Zhao M. et al., 2024). This discrepancy between the prevalence of infection and the rarity of carcinogenesis suggests that while HR-HPV is a necessary initiator, the loss of homeostasis in the host’s local microenvironment is the critical driver of malignant transformation. Therefore, deeply analyzing the regulatory mechanisms of the host microenvironment is of great clinical significance for early risk stratification and precision intervention.

Advancements in multi-omics have recently reshaped our comprehension of the cervical landscape, revealing that disease progression is inextricably linked to profound dysbiosis (Łaniewski et al., 2018). This dysbiosis is characterized by the depletion of protective dominant species such as *Lactobacillus crispatus*, and the dominance of anaerobic communities designated as community state type IV, represented by *Gardnerella*, *Mobiluncus*, *Sneathia*, *Megasphaera*, and *Atopobium (*Wu et al., 2023; Li Y. et al., 2025). This transition from *Lactobacillus*-dominance to anaerobe-dominance not only disrupts the local acidic physical barrier but also induces extensive metabolic remodeling. Dysbiotic microbiota induce the classic Warburg effect and lipid metabolism disorders in epithelial cells by producing high levels of short-chain fatty acids and biogenic amines (Li and Sui, 2021). Notably, the microbiota and host metabolism do not exist in isolation; they form complex bidirectional regulatory networks via metabolites, which not only directly regulate host signaling pathways but also shape an inhibitory immune microenvironment through immunometabolic reprogramming, driving immune escape and therapy resistance (Decout et al., 2024).

Although studies have largely examined either microbial diversity or isolated metabolic pathways, a systematic view of how the host–microbiota metabolic network drives carcinogenesis and treatment resistance across disease stages is still missing. Therefore, this review aims to summarize the dynamic evolution of the host–microbiota co-metabolic network from a healthy state to cervical cancer progression, analyzing the molecular mechanisms by which metabolites drive carcinogenesis through receptor activation, epigenetic regulation, and immunometabolic interactions. Furthermore, this paper will emphasize the core role of this network in promoting treatment resistance, providing new perspectives for overcoming clinical drug resistance and achieving personalized early warning and precision diagnosis and treatment.

## 2 Homeostatic construction of the cervical microbiota-host co-metabolic network

**Figure 1:**
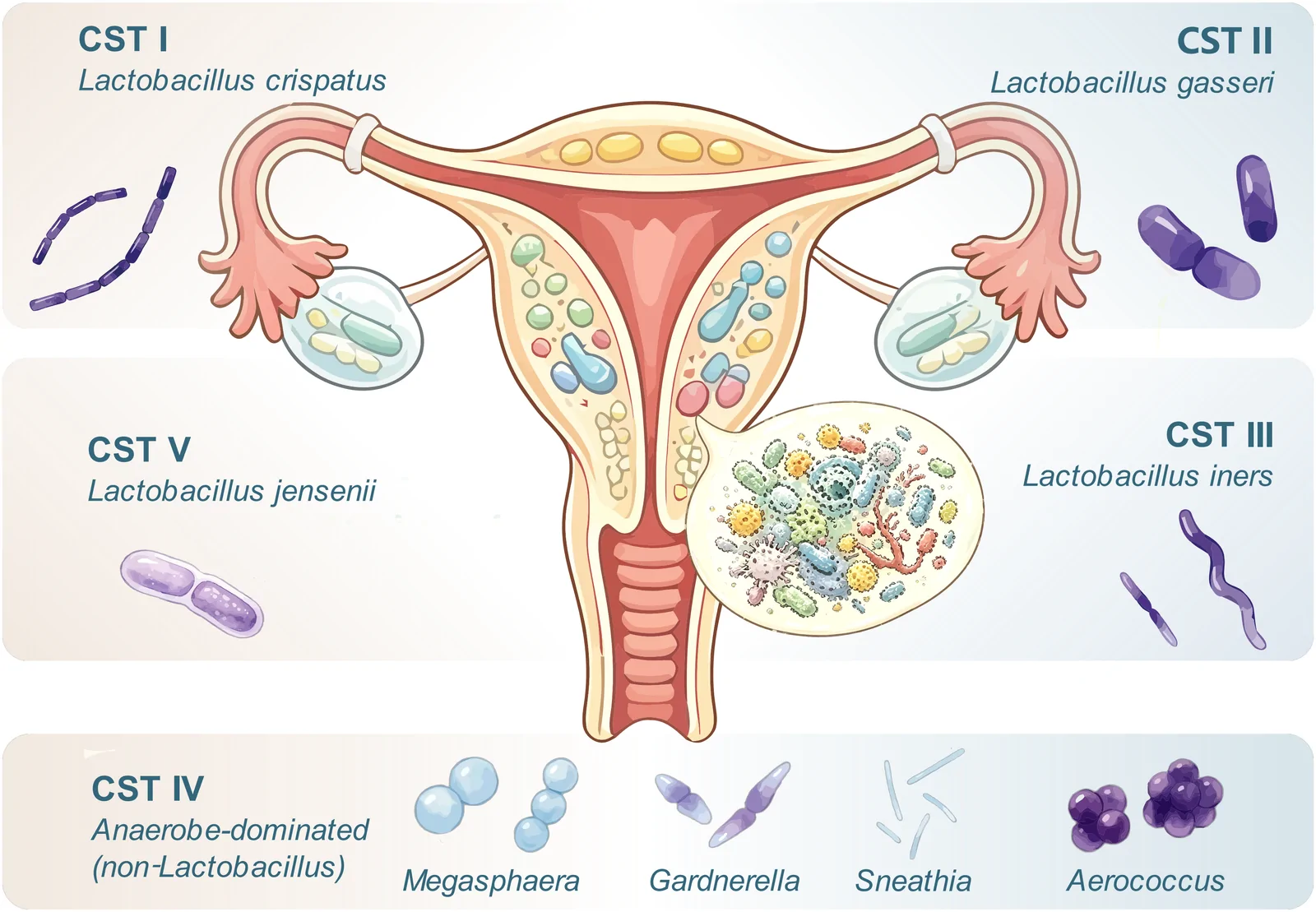
Cervicovaginal microbiome community state types. This image summarizes the five primary community state types of the cervicovaginal microbiome. CSTs I, II, III, and V are each dominated by different Lactobacillus species, which maintain a low, healthy pH. CST I (L. crispatus-dominant) demonstrates the highest stability and protective efficacy, whereas CST III (L. iners-dominant) is the most prevalent globally despite its ambiguous protective role. In contrast, CST IV is characterized by a high diversity of anaerobic bacteria, absence of Lactobacillus dominance, elevated pH, and association with bacterial vaginosis and adverse clinical endpoints.

The global distribution of human papillomavirus (HPV) demonstrates marked geographical heterogeneity. HPV16 and HPV18 are the predominant oncogenic types worldwide (Wei et al., 2024); however, the spectrum of high-risk genotypes varies considerably across regions. For instance, HPV58 is particularly prominent in China (Han et al., 2023), HPV52 is common in Japan (Konno et al., 2011), HPV35 and HPV45 are more frequently detected in Africa, whereas HPV31 and HPV51 prevail in several European countries (Zhou et al., 2024). In parallel, the vaginal microbiome composition also exhibits substantial regional variation. Among reproductive-aged women, community state type I (CST I), dominated by *Lactobacillus crispatus*, prevails in Europe and North America, whereas populations in sub-Saharan Africa (SSA) and Latin America more often show CST III (predominated by *L. iners*) or CST IV, a non-*Lactobacillus*-dominant state frequently enriched with anaerobic genera such as *Gardnerella*, *Prevotella*, and *Atopobium (*Roachford et al., 2022; Wang et al., 2023) (**Figure 1**). This geographical clustering of specific HPV genotypes and distinct microbial community types raises the possibility of an association between HPV epidemiology and the local cervical microenvironment.

A meta-analysis encompassing African and Asian populations further revealed that, among the general population, both vaccine-targeted and non-vaccine HPV genotypes exhibit significantly higher infection rates in SSA than in Asia (*p* < 0.001) (Okoye et al., 2021). Notably, SSA not only bears one of the highest global burdens of cervical cancer but also has the highest prevalence of bacterial vaginosis (approximately 55% of women) and the highest HIV-1 burden (accounting for 60% of global HIV infections) (Nyemba et al., 2022; Orlando et al., 2025). The concurrent occurrence of elevated HPV infection, pronounced vaginal dysbiosis, and immune deficiency in this region implies that the cervical microenvironment may have an underrecognized role in modulating HPV persistence and oncogenic progression.

**Figure 2:**
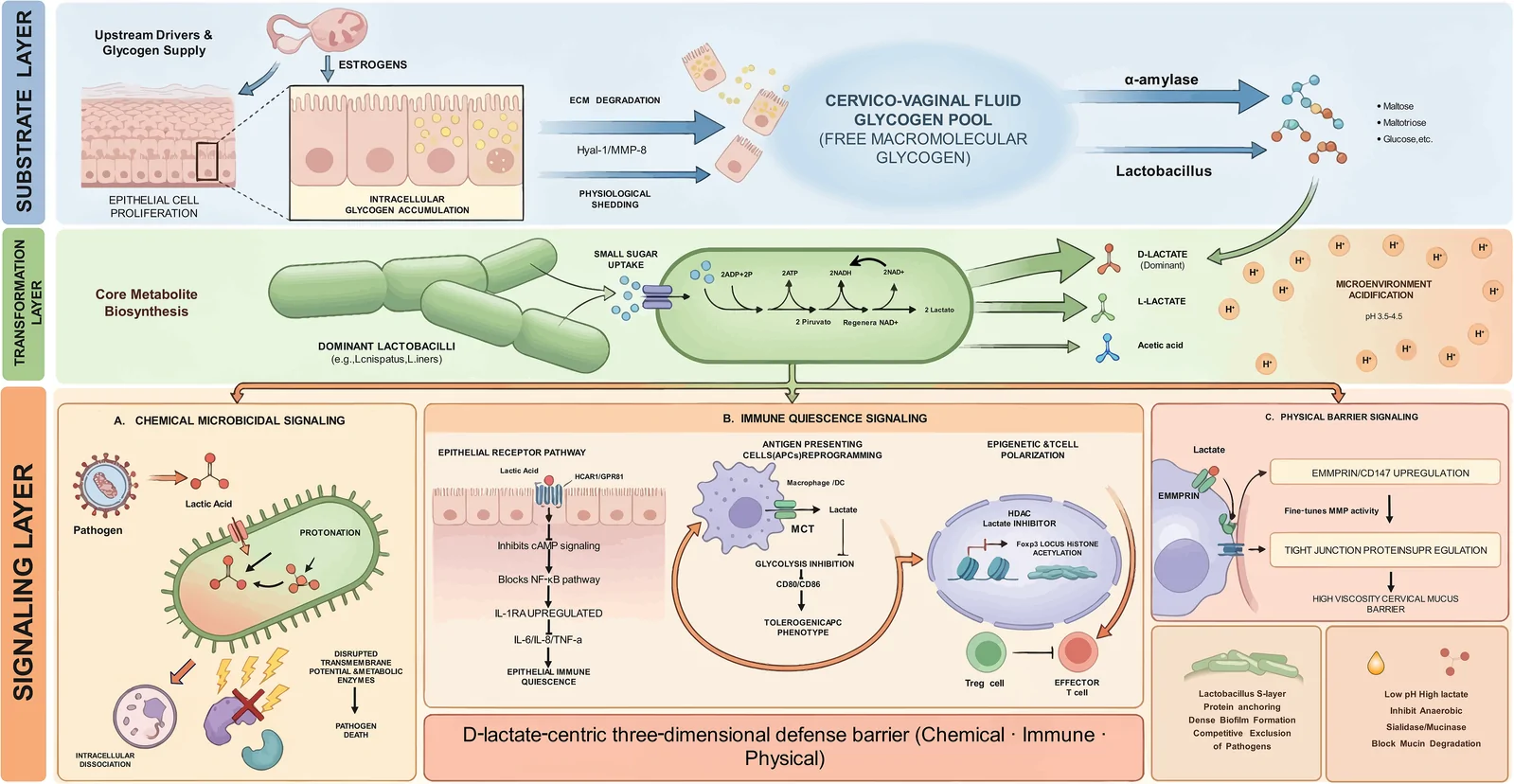
Homeostatic state: the cervical microbiota-host co-metabolic network. This schematic delineates the mechanistic basis for a D-lactate-centric defense system in the cervical mucosa. The process begins with estrogen-dependent glycogen deposition and liberation in the ​substrate layer, generating a nutrient pool for microbial metabolism. In the ​transformation layer, preferential sugar utilization by lactobacilli results in high-output D-lactate production and profound ambient acidification. The ​signaling layer​ integrates the effector functions of these metabolites: a ​chemical kill pathway​ mediated by proton influx, an ​immune tolerance pathway​ silencing epithelial, innate, and adaptive responses, and a ​barrier enhancement pathway​ fortifying mucosal integrity. Convergent actions of these pathways, aided by lactobacilli colonization, establish a robust, self-sustaining defensive homeostasis.

The cervical microenvironment is a complex ecosystem composed of the microbiota, the epithelial barrier, and the immune system. In this complex environment, microorganisms form close interdependent relationships with the host through the transformation of metabolites. To systematically analyze this homeostatic mechanism, this review proposes the concept of the “cervical microbiota-host co-metabolic network” and elaborates by decomposing it into three functional levels: the substrate layer, the transformation layer, and the signaling layer (**Figure 2**).

### 2.1 Substrate layer: nutrient supply mediated by the estrogen-glycogen axis

The substrate layer refers to the nutrients that microbiota depend on for surival and serves as the starting point of the co-metabolic network. The most abundant substrates in the local cervical environment are glycogen and mucin (Lacroix et al., 2020), whose metabolism and secretion are regulated by hormones and physiological cycles (Kaur et al., 2020), forming the primary metabolic interface of “host supply-microbiota utilization.”

Estrogen modulates the cervicovaginal microenvironment by promoting vaginal epithelial cell proliferation and glycogen synthesis. Glycogen is stored within epithelial cells, released into the lumen upon desquamation, and subsequently degraded by host-derived α-amylase, thereby making the resulting metabolites available to the microbial community (Jenkins et al., 2023). In accordance with this, α-amylase activity in cervical mucus is positively associated with Lactobacillus abundance. Meta-analyses of multiple cohort studies indicate that the activity of this regulatory axis shifts across life stages: transient Lactobacillus colonization occurs in neonates under the influence of maternal estrogen; estrogen levels rise during puberty and pregnancy, coinciding with the re-establishment of *lactobacilli* as the dominant taxa; and after delivery and menopause, declining estrogen levels are associated with reduced Lactobacillus abundance and a transition toward an anaerobe-enriched community structure (Ratten et al., 2021). By contrast, the effects of progesterone on the microbiota have been inconsistent across studies, and elevated testosterone levels may be associated with the more complex microbial profiles observed in patients with polycystic ovary syndrome(PCOS) (Jin et al., 2023). Collectively, these observations identify the estrogen-glycogen axis as a central regulatory node at the substrate layer.Beyond these endogenous hormonal rhythms, exogenous hormone administration also exerts modulatory effects. Oral contraceptives and other hormonal contraceptives have been associated with alterations in cervicovaginal microbial composition and mucosal inflammation, and long-term use has been associated with HPV persistence and cervical cancer risk; however, the specific microbial mediators involved remain to be clarified (Byrne et al., 2021; Serrano et al., 2025).

Autoimmune disorders and psychological stress constitute an additional dimension of hormonal influence on the cervicovaginal ecosystem. Chronic stress, acting through glucocorticoid-mediated immunosuppression and local inflammatory pathways, has been linked to abnormal vaginal discharge, epithelial disruption, and bacterial vaginosis, and may create a permissive milieu for HPV persistence (Rai et al., 2022). Autoimmune conditions themselves are also associated with an increased risk of malignant transformation (Gusakov et al., 2024).

Recent research reveals that glycogen is not an exclusive resource for *Lactobacillus*. Genes encoding glycogen-degrading enzymes are widely distributed across various Community State Type (CST) genomes, suggesting glycogen utilization by diverse microbial groups (Hertzberger et al., 2022; Jenkins et al., 2023). For instance, *Gardnerella* species possess sophisticated sugar transport systems that may influence their ecological niche within the cervical microenvironment (Bhandari and Hill, 2023; Nguyen et al., 2026). Furthermore, mucin serves as a nitrogen and complex carbon source whose degradation products are utilized by various taxa through cross-feeding, thereby further enriching the metabolic network of the substrate layer.

### 2.2 The transformation tier: lactic acid as the metabolic keystone

The transformation layer refers to the process where the microbiota converts substrates into primary metabolites, as well as the subsequent conversion of these metabolites by other microbial communities or host cells, serving as the engine of the co-metabolic network.

In a healthy state, the cervical microecology is dominated by the genus *Lactobacillus*, with key species including *Lactobacillus crispatus*, *L. jensenii*, and *L. gasseri *(Lagunas-Cruz et al., 2026). These beneficial bacteria convert glycogen-derived glucose into lactic acid through homofermentation, maintaining the cervicovaginal pH below 4.5 (Boskey et al., 1999). This acidic environment selectively inhibits the proliferation of anaerobic bacteria associated with bacterial vaginosis (BV) and is considered to reduce susceptibility to human immunodeficiency virus (HIV) and other sexually transmitted pathogens (Aldunate et al., 2013; Zhao et al., 2022).

Notably, the lactic acid metabolic capacity varies significantly among different *Lactobacillus* species. Microbiota dominated by *L. crispatus* produce high levels of D-lactic acid, while *L. iners* primarily produces L-lactic acid with a lower total output (Witkin et al., 2013). This metabolic difference directly affects cervical epithelial barrier function; *L. crispatus* supernatants can effectively attenuate barrier disruption induced by lipopolysaccharide or *Gardnerella vaginalis* and reverse the expression of inflammation-related microRNAs (Santarelli et al., 2025). In contrast, the protective effect of *L. iners* is significantly weakened due to its low lactic acid production and lack of D-lactic acid. Interestingly, although *L. iners* exhibits greater metabolic plasticity and is more easily replaced by pathogens during microenvironmental disturbances, it can still persist stably in some healthy women, suggesting that lactic acid metabolism is not the sole factor determining its function (Bhattacharya et al., 2023). Overall, *L. crispatus* constructs a lactic acid-centered metabolic barrier through the high production of D-lactic acid, acting as a key metabolic engine for maintaining cervical microenvironmental homeostasis.

Besides lactic acid, short-chain fatty acids (SCFAs) remain at low levels in the healthy state. Research shows that under physiological conditions mimicking a *Lactobacillus*-dominated environment, lactic acid maintains its anti-inflammatory effects even when coexisting with low concentrations of SCFAs (Schwecht et al., 2023). This contrasts with the pathological state of BV, where SCFAs are significantly elevated and exert pro-inflammatory effects. A hallmark of vaginal dysbiosis is the reduction of lactic acid and increased SCFA synthesis, with acetate being the primary metabolite in the cervicovaginal fluid of symptomatic BV patients (Delgado-Diaz et al., 2019). Therefore, the transformation layer constructs a core metabolic network to maintain microecological homeostasis through multiple mechanisms, including lactic acid metabolism (Delgado-Diaz et al., 2019), bacteriocin production (Scillato et al., 2021), adhesion competition (Osset et al., 2001), and microbial cross-feeding (Landolt et al., 2025). The functional differentiation and metabolic complementarity of different *Lactobacillus* species ensure the robust operation of this network, laying the metabolic foundation for immune regulation at the signaling layer.

### 2.3 Signaling layer: metabolite-mediated immune homeostasis

The signaling layer refers to the process in which metabolites act as signaling molecules to regulate host cell functions, revealing the functional transition of metabolites from metabolic intermediates to inter-kingdom signaling molecules.

Under healthy conditions, metabolites derived from *Lactobacillus* maintain local immune tolerance and epithelial barrier integrity through multidimensional mechanisms. Physiological concentrations of lactic acid have been shown to induce cervicovaginal epithelial cells to express IL-1RA and to inhibit the release of pro-inflammatory mediators, including IL-6, IL-8, and tumor necrosis factor-alpha (TNF-α), triggered by Toll-like receptor (TLR) agonists. This effect is not mimicked by hydrochloric acid at an equivalent pH and persists even after washing following lactic acid pretreatment, indicating that the lactic acid molecule itself possesses direct anti-inflammatory activity (Hearps et al., 2017). Furthermore, lactate is involved in immune tolerance through modulation of dendritic cell (DC) function (Sangsuwan et al., 2020). *Lactobacillus*-derived metabolites shift DCs toward an immature or tolerogenic state by suppressing antigen cross-presentation, modulating nuclear factor kappa-B (NF-κB) and GPR81 signaling, altering surface co-stimulatory and major histocompatibility complex (MHC) molecule expression, and promoting endogenous lactate production in tolerogenic DCs. Collectively, these changes reduce DC secretion of pro-inflammatory cytokines such as IL-12 and inhibit effector T-cell activation. This process serves to establish and maintain immune tolerance within the cervicovaginal mucosa and other local microenvironments, representing a key mechanism for preventing excessive inflammation and preserving tissue homeostasis (Sanmarco et al., 2023; Saito et al., 2024).

Short-chain fatty acids (SCFAs), present at low concentrations in the healthy state, are important signaling molecules that participate in immune tolerance. The metabolic network in the cervical microenvironment, which is largely driven by *Lactobacillus*, differs fundamentally from that governing intestinal mucosal immunity. In the gut, SCFAs such as butyrate support regulatory T cell (Treg) differentiation and sustain immune tolerance through histone deacetylase (HDAC) inhibition (Arpaia et al., 2013). In the healthy cervix, however, butyrate and propionate are scarcely detectable. Elevated butyrate levels are instead associated with bacterial vaginosis, a condition that compromises the epithelial barrier and elicits pro-inflammatory responses. In this setting, lactate assumes a functionally analogous role. At physiological concentrations, lactate enters immune cells via monocarboxylate transporters (MCTs), inhibits HDAC activity, increases histone acetylation at the *Foxp3* locus, and shifts naïve T cells toward a Treg lineage (Gaskarth et al., 2025; Lira et al., 2025). Beyond its epigenetic actions in immune cells, lactate also binds to the GPR81 receptor on cervical epithelial cells, suppressing the cAMP/NF-κB pathway and dampening the initiation of inflammatory signaling at the epithelial surface. Moreover, lactate enhances oxidative phosphorylation (OXPHOS) and represses glycolysis, skewing T cell differentiation toward an immunoregulatory phenotype (Bachem et al., 2025). Notably, trace acetate in the healthy cervix can weakly activate GPR43, but its signal is outweighed by the far greater concentration of lactate and does not dominate local immunomodulation.

In the cervical tumor microenvironment, microbial dysbiosis can reconfigure the metabolite landscape. Butyrate and other SCFAs may, on one hand, exert antitumor effects via HDAC inhibition, yet on the other hand, they might fuel tumor growth through metabolic reprogramming or foster immune escape by impairing effector T cell function (Wang et al., 2022). Consequently, whether a metabolite acts in an immune-protective or immune-evasive manner depends on its local concentration gradient, crosstalk within the metabolic network, and the broader context of the tumor immune microenvironment. Unraveling this complexity will require deeper investigation in future studies (**Table 1**).

**Table 1:** Metabolic signatures and host targets in progression from CIN to cervical cancer.

CIN stage	Metabolite category	Source	Host target/receptor	Core biological effect	Sample source	References
Early HPV infection/Homeostasis	Host defense peptide (HDP)	*Lactobacillus* spp.	mucosal innate immunity	HPV infection downregulates host mucosal antimicrobial peptides, limiting amino acid sources for Lactobacillus and driving dysbiosis	Cervicovaginal fluid, cervical tissues	(Lebeau et al., 2022)
CIN/Invasive CC	Antimicrobial peptide suppression (HBD2)	HPV16 E7 oncoprotein	ASK1-p38 MAPK pathway	HPV16 E7 inhibits HBD2 expression via ASK1-p38 downregulation, impairing epithelial innate immunity	Cervical tissues, cell lines	(Liao et al., 2025)
Early HPV infection	Glycogen metabolism	HPV-driven host response	Not specified	HPV infection significantly accelerates glycogen metabolism in cervical cells with large nuclei	Cervical epithelial cells	(Sitarz et al., 2020)
Invasive CC	Glucose/Glycolytic reprogramming	Host metabolic rewiring	p53, PI3K/AKT/mTOR, Wnt (overview)	HPV oncoproteins drive comprehensive metabolic reprogramming to support proliferation and immune evasion	NA (review)	(Gore et al., 2025)
Early HPV infection/Dysbiosis	Mucin glycoprotein degradation	*Prevotella bivia*, *Gardnerella vaginalis*	Cervical mucin glycoproteins	Sialidase-mediated mucin degradation disrupts epithelial barrier, facilitating HPV invasion	Cervicovaginal secretions/mucus	(Pelayo et al., 2024; Klimowicz et al., 2025)
Invasive CC	Lysophosphatidic acid (LPA)	Host-derived lipid mediator	LPA receptors 2/3 (LPA2/3)	LPA-mediated IL-8 secretion promotes angiogenesis in cervical cancer cells	Cervical tumor tissues	(Chen et al., 2012)
Invasive CC	Serine metabolism	Host SERINC2 upregulation	SERINC2; CD8+ T cells	SERINC2-mediated serine metabolism promotes CC progression and drives CD8+ T-cell exhaustion	Cervical tumor tissues	(Sun et al., 2025)
Invasive CC	L-Lactate (tumor-derived)	Cancer cell metabolism	RAS/PI3K oncogenic signaling axis	Lactate activates RAS and PI3K signaling; lactate oxidase disrupts this oncogenic pathway	Tumor tissues, cancer cell lines	(Keller et al., 2024)
Invasive CC (mechanistic model)	2-Hydroxyglutarate (2-HG)	Host/tumor metabolic intermediate	Histone demethylases; Rph1	2-HG modulates histone methylation at specific loci and alters gene expression (model system, not cervical-specific)	Cell models (non-cervical mechanistic study)	(Gavriil et al., 2024)
Invasive CC	Pentose phosphate pathway (G6PD lactylation)	HPV16 E6 oncoprotein	G6PD	HPV16 E6 activates the pentose phosphate pathway by inhibiting G6PD lactylation, promoting CC cell proliferation	Cervical cancer cell lines	(Meng et al., 2024)
Invasive CC	Aerobic glycolysis	lncRNA/host axis	NEAT1/miR-101-3p/RAC1 axis	NEAT1 regulates the miR-101-3p/RAC1 axis to affect aerobic glycolysis and CC progression	Cervical cancer tissues, cell lines	(Cao et al., 2025)
Early HPV clearance/Immune modulation	Tryptophan metabolite (Indole-3-lactic acid, ILA)	*Lactobacillus gasseri*	Aryl hydrocarbon receptor (AhR)	*L. gasseri*-derived ILA activates AhR, improving host immune response (viral clearance inferred but not directly proven)	Cervical epithelial cells	(Zhang Z. et al., 2025)
Invasive CC	Tryptophan–kynurenine–AhR axis	Host IDO1 enzyme	IDO1; Aryl hydrocarbon receptor (AhR)	High IDO1 expression increases kynurenine, which induces Treg differentiation via AhR, contributing to immune evasion and poor survival	Cervical tumor tissues	(Mezrich et al., 2010; Jayakumar et al., 2022)
CIN II–III/CC	Purine salvage pathway	Host systemic metabolism	HGPRT	Purine metabolic dysregulation is observed across circulation and tissues in pan-cancer analysis (extrapolated to cervical)	Plasma (pan-cancer extrapolation)	(Yu et al., 2025)
Invasive CC	MYC-driven oncogenic pathway	Host SERINC2	MYC pathway	SERINC2 drives CC progression through regulation of the MYC pathway	Cervical cancer tissues, cell lines	(Wang et al., 2024)

## 3 Dynamic imbalance of co-metabolic networks and carcinogenic drivers

### 3.1 Substrate level: competition and reorganization of metabolic resources

#### 3.1.1 Nutrient deprivation and resource hijacking

During the initial stage of HPV infection, the primary task of the virus is to create a microenvironment conducive to its own amplification. This metabolic shift is primarily executed through the subversion of host substrate supply, characterized by the systematic sequestration of carbon sources and the abrupt interruption of nitrogen availability.

This nitrogen restriction involves viral suppression of host defense peptides (HDPs), which function as immune barriers and also serve as nitrogen sources, supplying amino acids for *Lactobacillus* growth in the cervical niche (Lebeau et al., 2022). Emerging evidence suggests that HPV E7 disrupts this homeostatic balance through multifaceted signaling interference. Specifically, E7 suppresses HDP expression by inhibiting the NF-κB and Wnt/β-catenin pathways through the targeted disruption of NEMO, CK1, and β-TrCP, while simultaneously downregulating the ASK1-p38 MAPK pathway (Lebeau et al., 2022; Liao et al., 2025). This convergent suppression of antimicrobial peptides, including HβD2, S100A7, and Elafin, likely deprives *Lactobacillus* of essential amino acids and compromises the epithelial response to pro-inflammatory triggers such as TNF-α and LPS, thereby facilitating a shift in the microbial niche.

While depriving *Lactobacillus* of protein sources, HPV further cuts off the energy supply of commensal bacteria at the source by reshaping the host’s glucose metabolism axis. Normal cervical cells are rich in glycogen, which serves as the primary carbon source for *Lactobacillus* to produce lactic acid through fermentation and maintain an acidic microenvironment (Lacroix et al., 2020). Raman microspectroscopy studies have shown that HPV infection significantly accelerates glycogen consumption in cervical epithelial cells, particularly within large nuclear cell populations, leading to a decline in cytoplasmic glycogen levels at the onset of infection (Sitarz et al., 2020). This change is not a random resource shortage but rather metabolic reprogramming of host cells to meet the demands of viral amplification. HPV E6 targets p53 for proteasomal degradation, relieving the inhibition of glucose transporter type 1 (GLUT1) and activating the PI3K/AKT/mTOR axis (Gore et al., 2025). Such metabolic reprogramming may supply nucleotide precursors and ATP to support viral genome replication and reduce the availability of substrates within the local microenvironment.

Simultaneously, inflammation-induced mucin glycosylation patterns also influence the carbon and nitrogen sources available to the local microbiota. In persistent HPV infection, opportunistic pathogens such as *Prevotella bivia* and *Gardnerella vaginalis* secrete high levels of sialidases (Lin et al., 2022). These enzymes cleave glycoproteins within cervical mucus, disrupting the physical barrier and liberating monosaccharides and amino acids from mucins to support the expansion of anaerobic bacteria (Pelayo et al., 2024; Klimowicz et al., 2025). This sialidase-mediated degradation of the mucus barrier may coincide with events that promote viral entry into epithelial cells, potentially converting the local substrate milieu from a protective barrier into a nutrient-rich environment that favors pathogen proliferation. Collectively, these observations suggest that monitoring the metabolic substrate balance in the cervical microenvironment could provide prognostic information supplementary to viral load measurements, a possibility that warrants future prospective evaluation.

#### 3.1.2 Toxin accumulation and substrate reallocation

A characteristic metabolic hallmark of cervical intraepithelial neoplasia (CIN) progression is the localized reallocation of lipid resources (Pu et al., 2025). Notably, metabolomic profiling reveals a significant inverse correlation between glycerophospholipid abundance and the *Atopobiaceae* family within lesioned tissues, a depletion that may reflect increased phospholipid demand for rapid membrane biogenesis in proliferating cells (Jimenez et al., 2025). Concurrently, pathogens such as *Gardnerella* secrete phospholipases that catabolize host membrane lipids into lysophospholipids, modifying the local environment. Rather than functioning as mere degradative byproducts, these bioactive lipids activate G protein-coupled receptors and stimulate PI3K/AKT and MAPK/ERK signaling cascades (Chen et al., 2012). This metabolic-signaling nexus may accelerate the cell cycle and sustain anti-apoptotic signals required for disease progression (Sui et al., 2015).

Notably,amino acid metabolism is substantially rewired during cervical intraepithelial neoplasia (CIN) progression. Beyond their conventional roles in protein synthesis, certain amino acid-derived metabolites have emerged as key immunometabolic regulators. In the CIN1 microenvironment, cervical specimens reveal that *Lactobacillus* is a major determinant of the vaginal metabolome and is positively correlated with DL-indole-3-lactic acid (ILA) in HPV-positive women (Dai et al., 2025). Mechanistically, ILA derived from *Lactobacillus gasseri* LGV03 activates the aryl hydrocarbon receptor (AhR) in cervical epithelial cells, potentiating innate immune responses via increased secretion of IFN-α, IFN-β, and chemokines such as MIP-1α and MIP-1β (Zhang Z. et al., 2025). Studies in colorectal cancer models have further shown that ILA exerts anti-tumor effects through epigenetic modulation of CD8^+^ T cell immunity (Zhang et al., 2023), suggesting that analogous mechanisms may operate in the cervix and warrant further investigation. This ILA-AhR axis thus appears to establish an indole-based protective barrier that may limit early dysplastic progression. In contrast, progression to CIN2+ lesions is associated with enrichment of the *Atopobiaceae* family, including Atopobium. Metabolomic analyses have linked *Atopobium* to specific metabolites, including 9,10-DiHOME and α-linolenic acid, which have been proposed as potential biomarkers of HPV infection (Zhang et al., 2024). Moreover, *Atopobiaceae* have been implicated in lipid modulation, oxidative stress, inflammatory responses, and immune evasion. These alterations may converge on epigenetic reprogramming, potentially involving histone acetylation, to promote dysplastic progression (Jimenez et al., 2025). These immunometabolic alterations may promote dysplastic progression, although the precise mediators and their mechanistic contributions remain to be fully characterized. Collectively, the immunometabolic landscape shifts from a protective ILA-AhR axis in CIN1 to a pro-inflammatory and potentially pro-oncogenic state associated with *Atopobiaceae* enrichment in CIN2+, highlighting the stage-specific metabolic vulnerabilities that may be therapeutically exploitable.

The CIN stage is characterized by a robust activation of nucleotide metabolism, evidenced by the enrichment of metabolites like xanthine and hypoxanthine. Metabolomic analyses of cervical cancer cell lines have revealed purine salvage pathway activity in HPV-positive cells. Within this pathway, hypoxanthine-guanine phosphoribosyltransferase (HGPRT) converts hypoxanthine to inosine monophosphate (IMP) (Pappa et al., 2021), a reaction that supplies purine nucleotide precursors for DNA and RNA synthesis. Although HGPRT upregulation has been reported in diverse malignancies (Yu et al., 2025), direct evidence linking its activity specifically to nucleotide biosynthesis in cervical lesions remains scarce.Interestingly, current evidence points toward an age-dependent metabolic stratification: a significant positive correlation between *Atopobium* and xanthine is exclusive to younger patients with CIN2+ and is notably absent in the elderly (Kawasaki et al., 2025). This divergence underscores distinct metabolic drivers across the lifespan: whereas younger patients exhibit a proliferation-centric phenotype dominated by nucleotide biosynthesis, the metabolic landscape in older patients appears to shift toward inflammatory pathways.

#### 3.1.3 Metabolic exchange and resource predation

Upon progression to invasive cervical cancer (CC), malignant cells exhibit pronounced metabolic predation within the tumor microenvironment,a mechanism instrumental in facilitating immune evasion and distant metastasis. Beyond its fundamental role in proteosynthesis and nucleotide metabolism, serine serves as a central metabolic node governing one-carbon flux and lipid synthesis (Jia et al., 2023). Notably, clinical evidence indicates that the expression of Serine Incorporator 2 (SERINC2) correlates positively with disease severity (Sun et al., 2025). Up-regulation of this metabolic regulator accelerates intracellular serine flux in cancer cells, which drives the competitive depletion of extracellular serine and deprives the local microenvironment of nutrients essential for immune surveillance.Given that the activation and effector potency of CD8+ T cells necessitate an adequate supply of serine to fuel one-carbon and lipid metabolic pathways, this competitive consumption precipitates T-cell exhaustion through nutrient deprivation.

Furthermore, the functional role of folate metabolism undergoes a distinct shift; whereas earlier stages prioritize genomic stability, the invasive phase redirects these pathways toward accelerated proliferation and epigenetic reprogramming. Driven by *Gardnerella* and HPV co-infection, the up-regulation of folate biosynthetic genes accelerates DNA synthesis. This metabolic shift alters S-adenosylmethionine availability, thereby reshaping the host epithelial DNA methylation landscape and specifically driving the focal hypermethylation of key tumor suppressor genes (Tango et al., 2020; Li Y. et al., 2025).

### 3.2 The transformation layer: metabolic functional evolution and pathological drivers

#### 3.2.1 The waning of protective responses and the initiation of pro-carcinogenic processes

Under physiological conditions, cervical metabolic homeostasis is governed by D-lactate, primarily synthesized by *Lactobacillus crispatus*. Notably, metabolomic profiling indicates that HPV infection triggers a pathological shift in the enantiomeric landscape rather than a mere reduction in total lactate levels, manifested as a declining D-lactate to L-lactate ratio driven by *Lactobacillus iners* or facultative anaerobes (de Magalhães et al., 2022; Myeong et al., 2025). Beyond its acidic properties, D-lactate serves as a critical regulator by suppressing matrix metalloproteinase-8 (MMP-8) and its inducer extracellular matrix metalloproteinase inducer(EMMPRIN), thereby safeguarding extracellular matrix integrity (Witkin et al., 2013). Furthermore, it modulates Yes-associated protein 1 (YAP1) activity via the PI3K-AKT axis to curtail the self-renewal of cervical stem cells (Witkin et al., 2013). Conversely, L-lactate functions as a signaling molecule that can activate the hydroxycarboxylic acid receptor 1 (HCA1) receptor, thereby promoting cellular drug resistance and driving malignant remodeling of the tumor microenvironment (Wagner et al., 2017b; Keller et al., 2024). During dysbiosis, the overgrowth of pathogens such as *Gardnerella* not only disrupts D-lactate-mediated protective pathways but also mobilizes inflammatory and hyperplastic signaling through metabolic byproducts and structural components (Shvartsman et al., 2023). Crucially, the biological efficacy of these metabolites is inextricably linked to their delivery vehicles. L. crispatus-derived nano-sized extracellular vesicles (nCEVs), typically 60–80 nm in diameter, are enriched with D-lactate and instrumental in driving M2 macrophage polarization to facilitate mucosal healing and angiogenesis. By suppressing the biogenesis of these high-potency vesicles, HPV effectively dismantles local metabolic repair mechanisms, establishing a biochemical niche conducive to viral persistence (Peng K. et al., 2025).

The transition of the vaginal microbiota toward CST IV is accompanied by the accumulation of cytotoxic metabolic by-products within the microenvironment (Jung et al., 2025). Metabolomic analyses have revealed significant elevations in polyamines, including putrescine and cadaverine, in cervical cells infected with *Veillonella* species and in cervical carcinoma tissues (Liu et al., 2024). These alkaline metabolites act through dual mechanisms: they foster a niche conducive to anaerobic proliferation by elevating local pH and trigger the production of ROS in host cells. The resulting oxidative stress leads to direct DNA damage, thereby establishing a mutagenic milieu that facilitates viral genome replication and integration.

Recent multi-omics analyses of matched cervicovaginal secretions and cervical tissues reveal that local SCFA concentrations remain essentially unchanged during early HPV infection and CIN progression. Rather, these early intermediate stages exhibit a specific reprogramming of glycerophospholipid metabolism that coincides with the enrichment of particular anaerobes, as exemplified by the *Prevotella bivia*-PE(18:1/0:0)-HPV and *Fusobacterium periodonticum*-PI(40:6)-HPV metabolic axes (Pu et al., 2025). Notably, *Fusobacterium* is an oral pathogen, and its presence in the cervical microenvironment raises the possibility that the cervical tumor microbiome is shaped not only by local factors but also by microbial translocation or systemic dissemination originating from oral and other distant sites.In contrast, in cervical cancer patients, gut dysbiosis characterized by depletion of SCFA-producing taxa such as *Ruminococcus* and *Christensenellaceae* accompanies peripheral NK cell exhaustion and compromised anti-tumor immune surveillance (Klimov-Kravtchenko et al., 2025). Collectively, these findings delineate a stage-dependent evolution of the microbiota-host metabolic axis: early neoplastic lesions correlate with localized lipid-centric alterations, whereas advanced malignancy involves systemic SCFA deficiency and immune dysfunction.

#### 3.2.2 Oncogenic induction and metabolic reprogramming

During CIN progression, a key transformation involves the ectopic production of pro-oncogenic metabolites. In the context of persistent HPV infection, the enrichment of *Sneathia vaginalis* and specific *Escherichia coli* strains is associated with the generation of 2-hydroxyglutarate (2-HG) (Peng Y. et al., 2025). As a structural analog of α-ketoglutarate (α-KG), 2-HG competitively inhibits a range of dioxygenases, including histone demethylases and DNA hydroxymethylases (Gavriil et al., 2024). This local accumulation of 2-HG coincides with systemic epigenetic remodeling mediated by the host-microbe co-metabolic axis. Such remodeling is accompanied by widespread hypermethylation in the promoter regions of tumor suppressor genes, which is associated with arrested squamous differentiation and maintenance of a stem-like, undifferentiated proliferative state in pathological cells. However, direct experimental evidence specifically linking microbiota-derived 2-HG to this epigenetic cascade in cervical cancer remains limited and warrants further investigation.

High-risk HPV oncoproteins remodel the metabolic landscape of infected host epithelial cells. The E6 oncoprotein promotes ubiquitin-proteasome-mediated degradation of the tumor suppressor p53, relieving suppression of glucose transporter expression and enhancing glucose uptake within lesional cells (Jimenez et al., 2025). To manage this increased glucose influx, HPV E6 and E7 oncoproteins cooperate to augment the expression and transcriptional activity of c-MYC, which upregulates hexokinase 2 (HK2) (Hoppe-Seyler et al., 2017), a key rate-limiting enzyme that favors a metabolic shift from oxidative phosphorylation toward aerobic glycolysis. To counteract the heightened glycolytic flux and associated oxidative stress, HPV16 E6 also activates the pentose phosphate pathway by suppressing G6PD lactylation, facilitating the generation of reducing equivalents and supporting nucleotide biosynthesis (Meng et al., 2024; Wang et al., 2024). Collectively, these virus-mediated metabolic adaptations, together with the local accumulation of microbiota-derived oncogenic metabolites, are hypothesized to promote a microenvironment conducive to malignant progression from cervical intraepithelial neoplasia to carcinoma in situ.

#### 3.2.3 Dysregulated glycolysis and metabolic symbiosis

As the disease advances to invasive carcinoma, glycolytic flux increases substantially and becomes less responsive to canonical feedback inhibition. In CIN lesions, glycolytic enhancement remains largely dependent on microenvironmental hypoxia. In invasive cancer, however, the long non-coding RNA NEAT1 sequesters miR-101-3p, which releases RAC1 from inhibition and drives constitutive upregulation of *HK2* and *PKM2 *(Cao et al., 2025). This regulatory rewiring enables cancer cells to maintain a glycolytic preference irrespective of oxygen availability, with flux levels reaching several times those observed in CIN3 and showing limited sensitivity to local oxygen tension. Invasive carcinoma is also characterized by the establishment of intratumoral metabolic symbiosis. Within solid tumors, oxidative malignant cells in well-oxygenated regions and glycolytic cells in hypoxic cores engage in an L-lactate shuttle mediated by monocarboxylate transporter 1 (MCT1) and monocarboxylate transporter 4 (MCT4). L-lactate is secreted by hypoxic cells and commensal *Lactobacillus iners* via MCT4, then actively scavenged by oxidative counterparts via MCT1, where it is converted to pyruvate to sustain the tricarboxylic acid (TCA) cycle (Sun et al., 2020). This metabolic partitioning optimizes glucose utilization across the tumor mass. Evidence from cervical cancer tissues indicates that both MCT1 and MCT4 are overexpressed in invasive cervical carcinoma and high-grade CIN, supporting the relevance of this metabolic paradigm to the cervical microenvironment. Clinically, elevated LDHA expression correlates with increased recurrence risk and reduced survival in cervical cancer patients, supporting its utility as a prognostic indicator (Reyna-Hernández et al., 2022).

In parallel, HPV E6/E7-mediated activation of the PI3K/AKT/mTOR signaling cascade upregulates sterol regulatory element-binding protein 1 (SREBP-1) in cervical epithelial cells (Bossler et al., 2019). SREBP-1 in turn promotes the expression of key lipogenic enzymes, including fatty acid synthase (FASN) and acetyl-CoA carboxylase alpha (ACACA). This lipogenic reprogramming supports the elevated lipid requirements associated with membrane biogenesis in rapidly proliferating cancer cells. In cervical cancer, FASN overexpression correlates with lymph node metastasis and adverse clinical outcomes, and has been associated with metastasis promotion through cholesterol reprogramming and lymphangiogenesis (Du et al., 2022). Vascular endothelial growth factors C and D (VEGF-C and VEGF-D) are well-established mediators of lymphangiogenesis in various malignancies; however, their direct connection to FASN/SREBP-1-driven lipid reprogramming in cervical cancer remains unclear. Collectively, these observations suggest that HPV-driven upregulation of lipid synthesis may contribute to both metabolic adaptation and metastatic potential in cervical cancer cells, yet the underlying mechanisms, particularly those linking lipogenesis to lymphangiogenesis, merit further investigation.

### 3.3 Signaling layer: metabolic transduction and immunological remodeling

#### 3.3.1 The duality of antiviral signaling: from establishment to disruption

Under physiological conditions and during spontaneous HPV clearance, specific probiotic strains, most notably *Lactobacillus gasseri* LGV03, exhibit robust tryptophan metabolic activity and convert tryptophan into indole-3-lactic acid (ILA) (Zhang Z. et al., 2025). ILA serves as a ligand for the aryl hydrocarbon receptor (AhR) in cervical epithelial cells; its engagement of AhR triggers the canonical signaling cascade, which enhances transcription of type I interferons (IFN-α, IFN-β) and chemokines (MIP-1α, MIP-1β) (Jia et al., 2023). This ILA-AhR axis thus contributes to an antiviral immune state that may favor viral clearance.In contrast, persistent HPV infection correlates with a shift in tryptophan metabolic flux. As microbial ILA production declines, host-derived indoleamine 2,3-dioxygenase 1 (IDO1) activity increases, diverting metabolism toward the kynurenine pathway (Jayakumar et al., 2022). In cervical cancer cells, IDO1 drives the tryptophan-kynurenine-AhR axis, which has been associated with malignant phenotypes including proliferation, migration, and invasion (Zhang S. et al., 2025). Notably, although kynurenine also activates AhR, its downstream effects differ functionally from those of ILA: it promotes regulatory T cell differentiation and restricts effector T cell proliferation, effectively converting AhR signaling from an antiviral defense mechanism into an immunosuppressive program that supports viral immune evasion (Mezrich et al., 2010). The direct interplay between reduced microbial ILA production and enhanced IDO1 activity in the cervical microenvironment, however, remains insufficiently understood and warrants further investigation.

Beneficial microbiota, exemplified by Lactobacillus crispatus, orchestrate immune homeostasis through a dual-action mechanism: leveraging steric hindrance to preclude the association of pathogen-associated molecular patterns (PAMPs) with TLRs, while simultaneously engaging the anti-inflammatory receptor DC-SIGN on host antigen-presenting cells to suppress NF-κB signaling cascades (Decout et al., 2024). Such interactions are pivotal in shielding the local immune microenvironment from deleterious hyper-inflammation. In contrast, the HPV E7 protein interacts with the HTRA1 serine protease to activate the PINK1/Parkin axis and induce mitophagy in keratinocytes (Zhang B. et al., 2026). Through the clearance of dysfunctional mitochondria and the reduction of mitochondrial DNA and reactive oxygen species leakage into the cytosol, this process correlates with decreased type I interferon expression (de Magalhães et al., 2022). Additionally, HPV E7 has been reported to interfere with cGAS-STING signaling via other mechanisms in different cellular models, suggesting that E7-driven mitophagy may represent one of several strategies employed by the virus to evade innate immune surveillance and facilitate persistent infection.

Beyond its conventional role in acid-base homeostasis, lactate exerts profound regulatory functions as a signaling mediator. Quantitative analyses demonstrate that L-lactate and D-lactate both precipitate a 38%-63% elevation in nuclear DNA-dependent protein kinase catalytic subunit (DNA-PKcs) expression, a surge which corresponds notably with a 15%-36% attenuation in viral genomic integration (Wagner et al., 2021). This mechanism is orchestrated via the G protein-coupled receptor HCAR1, which triggers the PI3K/AKT signaling axis to upregulate DNA-PKcs transcription. By reinforcing the non-homologous end joining (NHEJ) repair pathway, lactate effectively biases viral DNA toward episomal maintenance, thereby precluding its integration into the host genome (Geris et al., 2023). Collectively, these observations suggest that lactate, through its modulation of host DNA repair pathways, may help counteract HPV-driven malignant progression.

The disruption of immune homeostasis is further compounded by bacterial constituents within the local microenvironment. Despite the absence of typical lipopolysaccharide (LPS), *Gardnerella* utilizes its cell wall components or secreted hemolysins to activate transcription factors such as NF-κB via the TLR/MD2 complex and a MyD88-dependent pathway, concurrently initiating MyD88- and TRIF-dependent collaborative signaling (Gardnerella vaginalis alters cervicovaginal epithelial cell function through microbe-specific immune responses, 2022). This triggers transcriptional cascades involving IRF3, NF-κB, and AP-1, leading to substantial secretion of pro-inflammatory mediators, notably IL-6 and IL-1β. These bacteria-driven inflammatory signals, in conjunction with HPV viral proteins, may activate the JAK/STAT3 signaling axis. Although STAT3 activation has been shown to promote PD-L1 expression through direct binding to response elements in the PD-L1 promoter in esophageal squamous cell carcinoma (Wang et al., 2025a), direct experimental evidence linking *Gardnerella vaginalis* to STAT3 activation and subsequent PD-L1 upregulation specifically within the cervical cancer microenvironment remains scarce. This proposed mechanistic axis therefore warrants dedicated future investigation.

#### 3.3.2 Persistent inflammation and immune checkpoint sequestration

Although interferon-stimulated genes (ISGs) provide an initial line of defense against HPV, the transition to the CIN stage is characterized by a sophisticated molecular synergy between *Gardnerella* and HPV16 that co-opts host interferon signaling. Mechanistically, *Gardnerella* exploits outer membrane vesicles to transport specific small RNAs or metabolites, thereby destabilizing the hsa-miR-654-5p-mediated competitive endogenous RNA (ceRNA) network (Şahin et al., 2023). This disruption accelerates the mRNA degradation of key antiviral transcripts, notably radical S-adenosyl methionine domain containing 2 (RSAD2) and interferon-induced protein with tetratricopeptide repeats 1 (IFIT1); the resulting depletion of these effector molecules effectively paralyzes the host’s ability to suppress viral replication. However, direct evidence linking *Gardnerella* vesicles to miRNA-mediated destabilization of RSAD2 and IFIT1 transcripts specifically within the cervical microenvironment remains to be established.

During the progression of CIN, high levels of succinate, produced by anaerobes like *Gardnerella* and *Prevotella*, engage the host G protein-coupled receptor GPR91. Binding triggers downstream signaling primarily via the Gαi subunit, activating phospholipase C (PLC) to induce IP3-dependent calcium mobilization and protein kinase C (PKC) activation. Succinate-GPR91 signaling has been implicated in inflammatory responses and NF-κB activation in various disease contexts (Zhang X. et al., 2026), and has been associated with the secretion of proinflammatory cytokines including IL-1β, IL-6, and TNF-α. Such an inflammatory milieu may favor HPV persistence and lesion progression, although the direct contribution of succinate-GPR91 signaling to cervical carcinogenesis remains to be defined.Distinct from the initial infection, within CIN lesions, cytokines secreted by tumor and stromal cells act predominantly through paracrine signaling on epithelial cells. This signaling activates the AKT and ERK pathways, leading to the upregulation of cyclin D1 and activation of the E2F transcription factor, which together drive cell cycle progression through the G1/S checkpoint into S phase. In HPV-positive cervical cancer, a virus-driven Rac1-NF-κB-IL-6 axis has been reported to activate STAT3 in an autocrine manner. IL-6 has also been linked to upregulation of carbonic anhydrase IX (CAIX), a protein associated with cervical cancer cell motility and disease progression (Morgan and Macdonald, 2019; Hsin et al., 2021). Collectively, these observations suggest that inflammatory signaling, particularly through the IL-6/STAT3 axis, may couple immune activation with metabolic reprogramming in cervical lesions. However, the precise molecular links connecting succinate-GPR91 signaling to the IL-6/STAT3/CAIX axis specifically within the cervical cancer microenvironment have not been directly established and warrant further investigation using cervical tissue-derived models.

Concurrently, colonization by *Atopobiaceae* family members establishes a metabolic axis that may sustain chronic inflammatory signaling within the cervical microenvironment. Metabolomic profiling has linked *Atopobiaceae* enrichment to pro-carcinogenic metabolites, including 4-hydroxybutyrate and sphingosine (Jimenez et al., 2025). Sphingosine functions as a damage-associated molecular pattern that activates the NLRP3 inflammasome, and mitochondrial reactive oxygen species (mtROS) serve as known triggers of both NLRP3 inflammasome activation and NF-κB signaling (Liang et al., 2021). Given that *Atopobiaceae*-rich profiles correlate with pro-inflammatory cytokines such as IL-1α and TNF-α (Saito et al., 2024), it is plausible that *Atopobiaceae*-derived lipid metabolites initiate a cascade involving mtROS overproduction, NLRP3 inflammasome activation, and NF-κB-driven inflammatory cytokine secretion. This pro-inflammatory milieu may in turn favor lesion development.Beyond these immediate inflammatory effects, the same microenvironment may engage cytokine-driven autocrine loops that reinforce malignant transformation. In HPV-positive cervical cancer, a virus-driven Rac1-NF-κB-IL-6 axis activates STAT3 in an autocrine manner, and IL-6-induced STAT3 activation is known to upregulate PD-L1 transcription across multiple cancer types. Notably, *Atopobiaceae* abundance exhibits a robust positive correlation with the expression of immune checkpoint ligands, including PD-L1 and LAG-3. Within this dysbiotic microenvironment, sustained elevation of IL-6 facilitates JAK-mediated STAT3 phosphorylation; the resulting p-STAT3 dimers translocate to the nucleus to drive PD-L1 transcription, effectively enabling lesion cells to evade T-cell-mediated surveillance (Jimenez et al., 2025). Although direct experimental evidence specifically connecting *Atopobiaceae* metabolites to these signaling cascades in the cervical microenvironment remains limited, the available data suggest a potential integration of inflammatory, immune checkpoint, and metabolic reprogramming pathways that warrants further investigation.

With escalating lesion density, the local microenvironment invariably transitions into a state of hypoxia. Evidence suggests that in the CIN3 stage, Hypoxia-Inducible Factors (HIFs) and their downstream effector, CAIX, undergo significant upregulation (Lee et al., 2008). By localizing to the cell surface, CAIX maintains a preferentially alkaline intracellular milieu, which facilitates sustained glycolytic flux. This resultant pH gradient drives extracellular acidification, which in turn activates proteases that compromise matrix integrity, creating conduits for viral entry and cellular migration (Benej et al., 2020; Hsin et al., 2021; Temiz et al., 2021). Simultaneously, the alkaline intracellular environment optimizes the kinetics of biosynthetic enzymes, thereby driving the rapid proliferative expansion of CIN lesions.

#### 3.3.3 Establishment and solidification of pro-oncogenic signaling networks

The transition to invasive carcinoma frequently coincides with a profound disruption of the epigenetic regulatory network, affecting roughly 30% of epigenetic modulators, including DNA methyltransferases, histone deacetylases, and chromatin remodeling subunits (Sisodiya et al., 2025). This widespread epigenetic instability is considered a hallmark of disrupted genomic homeostasis. Within this context, the HPV E7 oncoprotein binds to DNA methyltransferase 1 (DNMT1) through its CR3 zinc-finger domain and upregulates DNMT3B expression, changes that correlate with aberrant hypermethylation of CpG islands in tumor suppressor gene promoters (Rosendo-Chalma et al., 2020). One illustrative example is the promoter of C-X-C motif chemokine ligand 14 (CXCL14), which is frequently hypermethylated in invasive lesions, a modification linked to its transcriptional silencing (Cicchini et al., 2016). Given the essential role of CXCL14 in recruiting natural killer cells and dendritic cells, its epigenetic repression may impair immune cell trafficking into the tumor site, a process that may contribute to the formation of an immune-excluded microenvironment.

During progression to invasive carcinoma, L-lactate accumulates within the tumor microenvironment and functions as a signaling molecule that influences both cell-intrinsic therapeutic resistance and extrinsic immune evasion. Mechanistically, L-lactate activates the hydroxycarboxylic acid receptor 1 (HCAR1/GPR81), which couples to Gi proteins to inhibit adenylate cyclase, with subsequent reduction in intracellular cAMP levels and engagement of the PI3K/AKT signaling axis (Wagner et al., 2017a). This signaling cascade has been associated with AKT-mediated phosphorylation of mouse double minute 2 homolog (MDM2), which enhances its E3 ubiquitin ligase activity and promotes p53 degradation. Concurrently, phosphorylation of Forkhead box O (FOXO) transcription factors is linked to reduced pro-apoptotic signaling (Wang et al., 2025b). These coordinated events may collectively enhance DNA damage repair mechanisms, potentially enabling malignant cells to resist cisplatin- and ionizing radiation-induced DNA double-strand breaks.Beyond its role in metabolic signaling, L-lactate also functions as an epigenetic modifier through p300-catalyzed histone lactylation, particularly at H3K18la (Liu R. et al., 2025). This post-translational modification influences the immunological landscape by promoting macrophage polarization toward a pro-tumorigenic M2 phenotype, characterized by upregulation of *Arg1*, *IL-10*, and *Mrc1*. In addition, histone lactylation has been linked to HIF-1α stabilization through inhibition of prolyl hydroxylase-mediated degradation, and HIF-1α has been associated with PD-L1 expression and reduced T-cell-mediated cytotoxicity (Li C. et al., 2025). It is worth noting that much of the evidence for these lactate-driven epigenetic and signaling cascades has been generated in glioma, pancreatic, and lung cancer models; direct validation in the cervical cancer microenvironment remains an open question.

**Figure 3:**
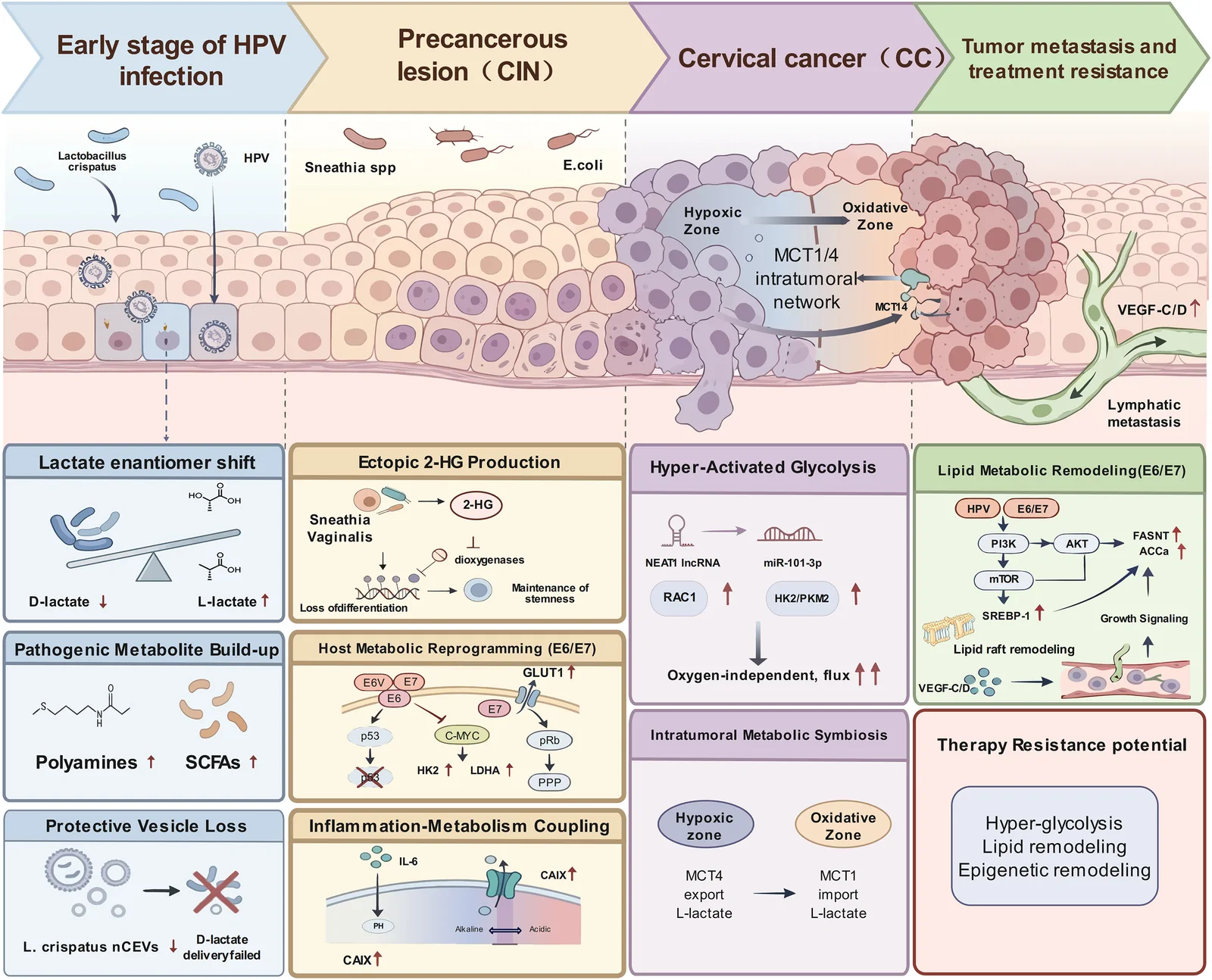
Metabolic evolution of signaling layers in the progression from HPV infection to cervical cancer. This image chronologically illustrates the key metabolic evolution during the progression from initial HPV infection to cervical cancer (CC). The process begins with early alterations post-HPV infection, including lactate enantiomer shift, accumulation of pathogenic metabolites, and reduction of protective vesicles, which collectively contribute to the development of cervical intraepithelial neoplasia (CIN). The CIN stage is further characterized by ectopic oncometabolite generation, host metabolic reprogramming, and inflammatory metabolic coupling, driving the progression to invasive carcinoma. Ultimately, cancer cells establish a self-sustaining promalignant metabolic factory, marked by dysregulated glycolysis, metabolic symbiosis, and polarized lipid metabolism.

Within the tumor microenvironment, lactate derived from glycolysis is exported by tumor cells expressing high levels of MCT4 and subsequently imported by tumor-infiltrating T cells via MCT1 (Peralta et al., 2024). This intracellular lactate accumulation may impair T cell function through multiple mechanisms, including disruption of lactate efflux, intracellular acidification, and perturbation of energy metabolism. These effects have been associated with suppression of anabolic pathways such as AKT/mTOR and activation of catabolic pathways including AMPK, changes that may ultimately reprogram T cell metabolism and contribute to functional exhaustion (Saravia et al., 2020). Although high lactate levels are associated with a therapy-refractory phenotype, this metabolic landscape is not strictly unidirectional. Notably, the persistence of *Lactobacillus crispatus* in the local niche has been associated with counteracting tumor progression; erucic acid secreted by these commensals activates PPARδ signaling and may trigger excessive fatty acid oxidation (FAO). The resulting increase in mitochondrial reactive oxygen species has been reported to selectively induce ferroptosis in cervical cancer cells, revealing a potential metabolic vulnerability within the tumor microenvironment that may offer therapeutic opportunities (Zhen et al., 2025) (**Figure 3**).

## 4 Co-metabolic dysregulation underpinning therapeutic resistance in cervical cancer

As a malignancy profoundly contingent upon microenvironmental stability, cervical cancer exhibits therapeutic responses shaped by a complex interplay between host genomic heterogeneity and microbial metabolic governance. Under therapeutic pressure, the relatively balanced “microbiota-host co-metabolism” becomes dysregulated. It transitions from a physiological state supporting local pH balance and immune tolerance to a pathological program that fosters tumor survival, immune evasion, and therapy resistance. This co-metabolic rewiring establishes a multifaceted resistance network via the direct action of metabolites, the remodeling of the host epigenome, and the formation of biological barriers.

### 4.1 Microbial features of treatment failure

Treatment failure in cervical cancer is associated with specific dysbiotic configurations of the local microbiota. A prospective longitudinal study of 36 chemoradiation (CRT) patients, reported that patients retaining *Lactobacillus crispatus* had a 3-year progression-free survival of 71%, whereas those who lost this species early had a rate of 39% (HR 0.43, *p* = 0.003) (Wu et al., 2025). In agreement with this finding, multiple studies have shown that higher baseline abundance of *L. crispatus* correlates with reduced recurrence risk. By contrast, elevated abundance of *Lactobacillus iners* above a certain threshold has been associated with poor treatment outcomes (Colbert et al., 2023). Unlike the protective role attributed to *L. crispatus*, *L. iners* is an obligate L-lactate-producing bacterium that may influence tumor metabolism and lactate signaling pathways, potentially contributing to therapeutic resistance. This lactate accumulation has been linked to HCAR1-mediated signaling, a pathway implicated in enhanced DNA damage repair and PD-L1 upregulation in other cancer models. Furthermore, a network of anaerobic pathogens, including *Fusobacterium nucleatum, Sneathia sanguinegens*, and *Atopobium vaginae*, has been associated with cervical lesion progression and may represent potential biomarkers for treatment outcomes (Peng Y. et al., 2025). However, the precise mechanisms through which these microbial taxa influence therapy response in cervical cancer remain to be fully elucidated.

These static baseline profiles capture the pre-treatment risk landscape, yet dynamic shifts in the microbiota during therapy reveal divergent trajectories between responders and non-responders. The acute phase of CRT typically causes a sharp reduction in L. crispatus in both groups, reflecting broad antimicrobial effects. Responders, nevertheless, display a distinct capacity for microbial restructuring, for example through expansion of radiation-tolerant *Lactobacillus gasseri*, which sustains lactate production and a low-pH milieu that may support local immune surveillance. In contrast, non-responders follow a different path: *L. iners* proliferates rapidly after radiotherapy, correlating with a steep rise in intratumoral lactate that coincides with HCAR1 activation and augmented DNA repair (Johnston and Bullman, 2024). During the subacute phase, non-responders also exhibit an increase in radiation-resistant Enterobacteriaceae, a shift that may be facilitated by L. *iners*-mediated epithelial barrier disruption and local lactate accumulation. Microbial alpha-diversity declines markedly in non-responders but remains relatively stable in responders. Notably, recovery of microbial diversity at 6 months post-treatment is associated with a lower risk of recurrence. Patients who re-establish *L. crispatus* dominance show improved survival compared to those with persistent dysbiosis (Du et al., 2025). These observations suggest that post-treatment restoration of microbial diversity may serve as a marker of local microenvironmental recovery and as an independent predictor of long-term outcome.

### 4.2 Metabolic reprogramming and chemoradioresistance

The intratumoral microbiota undergoes notable shifts during chemoradiotherapy for advanced cervical cancer. In patients with cervical cancer, colonization by *Lactobacillus iners* has been associated with poorer survival outcomes, and genomically related lactate-producing bacteria have been linked to reduced survival across multiple solid tumor types (Colbert et al., 2023). At the cellular level, L-lactate produced by *L. iners* has been reported to induce metabolic reprogramming in cervical tumors. In cervical cancer cell lines, lactate uptake through monocarboxylate transporters stabilizes hypoxia-inducible factor 1α (HIF-1α), a transcription factor that has been associated with upregulation of glycolytic enzymes and suppression of oxidative phosphorylation. In other cancer models, lactate has been linked to nucleotide synthesis and DNA repair through metabolic reprogramming (Wang et al., 2026), and lactate-derived pyruvate may fuel the tricarboxylic acid cycle to sustain ATP production under glucose-limited conditions, raising the possibility that analogous mechanisms operate in lactate-rich cervical tumors.Beyond its cell-intrinsic effects, lactate may also influence the tumor microenvironment. In various cancer types, lactate has been associated with activation of cancer-associated fibroblasts (CAFs), which then secrete hepatocyte growth factor (HGF) and stimulate tumor cells to release fibroblast growth factor 2 (FGF2) and transforming growth factor-beta (TGF-β) (Linares et al., 2022). These signals may collectively promote the formation of a fibrotic, immunosuppressive extracellular matrix that has been linked to impaired drug penetration, worsened hypoxia, and a therapy-refractory phenotype. While CAFs have been implicated in cervical cancer progression (Qu et al., 2025), the specific contribution of lactate-driven CAF activation to therapeutic resistance in this context requires further investigation.

Microbially derived lactate influences radio- and chemoresistance through distinct but convergent pathways that modulate DNA damage repair. One such pathway involves lactate binding to the hydroxycarboxylic acid receptor 1 (HCAR1/GPR81). In cervical cancer cell lines, HCAR1 activation has been associated with enhanced DNA repair capacity and resistance to chemotherapeutic agents. HCAR1 silencing has also been linked to reduced expression of the ATP-binding cassette subfamily B member 1 (ABCB1) transporter, correlating with increased intracellular drug accumulation in HeLa cells.In addition to receptor-mediated signaling, lactate functions as an endogenous HDAC inhibitor in cervical cancer cells, inducing histone hyperacetylation and upregulating DNA repair genes including *LIG4*, *NBS1*, and *APTX* (Wagner et al., 2015). This epigenetic modulation has been correlated with accelerated DNA repair and reduced persistence of radiation-induced DNA damage (Ciszewski et al., 2022). More recently described layer involves lactate-derived histone lactylation, a novel epigenetic modification that influences chromatin accessibility and the assembly of DNA repair complexes. In gastric cancer models, lactylation of *NBS1* has been linked to efficient DNA repair and chemotherapy resistance (Chen et al., 2024). In cervical cancer, *NDRG1* has been identified as a driver of radioresistance through homologous recombination-mediated DNA repair (Yu et al., 2024), and histone lactylation has been proposed as a potential upstream regulator of *NDRG1* transcription. Collectively, these lactate-associated modifications may contribute to a persistent therapy-resistant phenotype; however, the complete cascade, particularly the histone lactylation-*NDRG1* axis, requires direct validation in cervical cancer models.

Tumor-intrinsic metabolic enzymes also contribute to glycolytic reprogramming. Proteasome activator complex subunit 3 (PSME3), which is overexpressed in cervical cancer, upregulates poly(ADP-ribose) polymerase 1 (PARP1) via c-Myc and has been linked to enhanced DNA damage repair and radioresistance (Wei et al., 2023); PSME3 silencing has been associated with suppressed glycolysis and impaired metabolic fitness. The long non-coding RNA UCA1 is elevated in radioresistant cells, where it enhances glycolytic flux through increased HK2 expression, an effect that is reversed by glycolytic inhibition (Wu et al., 2018). UDP-glucose ceramide glucosyltransferase (UGCG) activates the PI3K/AKT pathway and correlates with increased glucose uptake and lactate output (Zhang and Zhang, 2021). HPV oncoproteins, notably E6 and E7, have been associated with enhanced glycolytic activity and chemoresistance, and inhibition of these oncoproteins has been linked to reversal of this phenotype.Beyond tumor-cell autonomous effects, tumor glycolysis and microbial metabolism engage in bidirectional crosstalk. Within this co-metabolic network, not all microbial activities promote resistance; certain beneficial taxa and their metabolites have been associated with improved treatment response, potentially through sustaining host homeostasis. For example, metabolites derived from *Bifidobacterium* scavenge free radicals and may alleviate radiation-induced intestinal mucositis, which may facilitate the delivery of full therapeutic radiation doses and enhance tumor cell killing. Clinically, microbial signatures enriched in *Bifidobacteriaceae*, *Kitasatosporaceae*, and *Orbaceae* have been associated with radiotherapy response, suggesting that the abundance of beneficial intratumoral bacteria may serve as an independent biomarker of treatment outcome (Dou et al., 2024).

### 4.3 Immunometabolism and resistance to immunotherapy

Intratumoral microbial metabolites, particularly lactate, have been implicated in both radioresistance and immune evasion. Lactate promotes lactylation at lysine 280 (K280) of PD-L1, a modification that impairs binding by the E3 ligase HUWE1 and subsequent ubiquitination, which has been associated with reduced PD-L1 proteasomal degradation. This modification has been linked to increased PD-L1 expression on tumor cells and has been associated with immune evasion (Liang et al., 2026). The resulting lactate-acidified tumor immune microenvironment (TIME) has been correlated with suppressed glycolysis and cytotoxic function in effector T cells, as well as with T-cell exhaustion (Ashok et al., 2024). This immunosuppressive state is particularly pronounced in patients with HIV co-infection.

Gut-derived metabolites can access the cervical mucosal microenvironment via the portal and systemic circulations, a physiological route that provides a rationale for considering systemic microbial influences in this local context. Among these circulating metabolites, short-chain fatty acids (SCFAs) and tryptophan catabolites have been shown to modulate systemic inflammatory tone and influence the metabolic fitness and effector functions of circulating and tissue-resident natural killer (NK) cells. This systemic-to-local signaling axis provides a mechanistic basis for examining whether gut dysbiosis contributes to immune evasion in cervical cancer. Indeed, HIV-associated gut dysbiosis has been linked to immunotherapy resistance through interconnected metabolic disruptions. Butyrate deficiency has been associated with impaired epithelial energy metabolism and compromised antitumor immunity. Dysregulated tryptophan metabolism, characterized by depletion of *Akkermansia muciniphila* and *Enterococcus*-driven overproduction of phenethylamine, has been correlated with compromised intestinal barrier integrity. Additionally, defective vitamin B synthesis has been associated with impaired DNA repair and disruption of the Th1/Th2 immune balance. Collectively, these perturbations have been linked to activation of NF-κB/STAT3 signaling and a chronic inflammatory state involving IL-6 and TNF-α, which has been associated with reduced effectiveness of immunotherapy (Meng et al., 2025).

The relevance of these systemic metabolic disturbances to cervical cancer immunity is supported by emerging evidence linking gut microbiota alterations directly to NK cell exhaustion in this disease. Clinically, NK cells in cervical cancer patients often display an exhausted phenotype, with upregulated inhibitory receptors including PD-1 and LAG-3. A significant positive correlation has been reported between gut dysbiosis indices and NK cell exhaustion scores. Specifically, cervical cancer patients exhibit gut dysbiosis characterized by enrichment of pro-inflammatory taxa such as Escherichia-Shigella and depletion of SCFA-producing bacteria including Ruminococcus and Christensenellaceae (Klimov-Kravtchenko et al., 2025). Given the established immunomodulatory roles of SCFAs such as butyrate in influencing NK cell phenotype and function (Carlini et al., 2025), it is plausible that reduced SCFA availability may influence NK cell activity, although direct evidence linking butyrate to NK cell metabolic support, such as oxidative phosphorylation, within the cervical cancer context remains lacking. Machine learning analyses have further indicated that dysbiosis scores can predict NK cell exhaustion, suggesting that gut microbial imbalance may influence anti-tumor immune surveillance. Additionally, systemic inflammation, a well-recognized contributor to immunosuppression (Sacdalan and Lucero, 2021), may parallel these microbiota-associated immune alterations, though integrative studies are needed to establish direct connections.

It should be noted, however, that these observations remain largely correlative; longitudinal cohort or intervention studies are required for validation, and direct mechanistic evidence for gut-to-genital tract signaling in cervical cancer is still lacking. Accordingly, microbiota-targeted strategies aimed at reversing NK cell exhaustion currently require stronger mechanistic foundations before clinical translation can be considered.

Within this immunometabolic landscape, ferroptosis has emerged as a metabolic vulnerability that may be exploited to counteract resistance. Ferroptosis, a metabolic form of cell death dependent on lipid peroxidation, has been proposed as a target for overcoming drug resistance, although a dysregulated co-metabolic network often poses a barrier. For example, exosomes derived from tumor-associated macrophages (TAMs) have been associated with downregulation of ALOX15, which has been linked to reduced unsaturated fatty acid peroxidation and ferroptosis resistance (Luo et al., 2023). In contrast, health-associated metabolic partners such as *Lactobacillus crispatus* have been associated with sensitization to ferroptosis; erucic acid produced by this bacterium activates PPARδ signaling and has been linked to a reactive oxygen species burst that may re-engage ferroptosis in drug-resistant cells, as demonstrated in organoid models (Zhen et al., 2025). Furthermore, targeting the PIN1/NRF2/GPX4 axis has been associated with enhanced cisplatin cytotoxicity, suggesting a potential strategy to reverse treatment resistance (Zhang et al., 2022) (**Table 2**).

**Table 2:** Key metabolites and effectors contributing to cervical cancer treatment failure and progression.

Source	Key metabolite/effector	Host pathway/target	Clinical/biological context	Evidence type	References
*Lactobacillus iners* (intratumoral bacteria)	L-Lactate	Metabolic reprogramming/Enhanced DNA repair	Locally advanced CC chemoradiotherapy resistance	Direct	(Colbert et al., 2023)
Host cervical cancer cells (HeLa)	L-Lactate	HCAR1 (GPR81)/ABCB1 transporter	CC chemotherapy (doxorubicin) resistance	Direct (HeLa)	(Wagner et al., 2017a)
Host cells	L-Lactate, D-Lactate	HCAR1 activation + HDAC inhibition	CC chemotherapy resistance (enhanced DNA repair)	Direct (CC cell lines)	(Wagner et al., 2015)
Host tumor microenvironment/Tumor cells	Lactate, Short-chain fatty acids (SCFAs)	DNA repair enhancement	CC chemoresistance & HPV infection modulation	Direct (CC cells)	(Ciszewski et al., 2022)
Host tumor cells	Lactate	NBS1	Pan-cancer chemoresistance (mechanism applicable to CC)	Indirect	(Chen et al., 2024)
Host cervical cancer cells	Lactate	PARP1/Aerobic glycolysis	CC radiotherapy resistance	Direct (CC tissues/cells)	(Wei et al., 2023)
Host cervical cancer cells	Oxidative stress/Ferroptosis-related	PIN1/NRF2/GPX4 axis	CC cisplatin chemosensitization	Direct (CC cell lines)	(Zhang et al., 2022)
*Gammaproteobacteria* (other tumor model)	Cytidine deaminase (non-lactate)	Gemcitabine metabolic inactivation	Other tumor (pancreatic cancer) chemoresistance	Other tumor	(Geller et al., 2017)
*Porphyromonas gingivalis* (intratumoral bacteria)	FimA virulence factor (non-metabolite)	CD151/ITGB1 (Integrin signaling)	CC distant metastasis (lymphatic/hematogenous)	Direct (CC tissues/models)	(Huang et al., 2025)
Gram-negative intratumoral bacteria (genus unspecified)	LPS (Lipopolysaccharide, bacterial component)	TLR4/MAPK signaling pathway	CC lymphatic metastasis	Direct (CC tissues/models)	(Liu Y. et al., 2025)
Host	Lactate (aerobic glycolysis)	miR-493-5p/HK2 axis	CC progression	Direct (CC cell lines)	(Wu et al., 2018)
Host (UDP-glucose ceramide glycosyltransferase)	Glycolysis	PI3K/AKT pathway	CC proliferation and metabolic reprogramming	Direct (CC cell lines)	(Zhang and Zhang, 2021)

### 4.4 Drug metabolism and physical barriers

Specific bacterial enzymes can directly modify anticancer drugs, an observation made across multiple cancer models. In pancreatic ductal adenocarcinoma, for example, intratumoral Gammaproteobacteria expressing a long isoform of cytidine deaminase (CDD_L) inactivate gemcitabine through conversion to a non-toxic uridine derivative, a process that has been linked to chemoresistance (Geller et al., 2017). Notably, *Gammaproteobacteria* have also been detected in the cervical cancer microenvironment, particularly following radiotherapy, suggesting that similar drug-inactivating mechanisms may operate in cervical tumors. In addition, gut microbial β-glucuronidase (GUS) can reactivate the detoxified irinotecan metabolite SN-38G to its toxic form SN-38, which has been associated with dose-limiting diarrhea (Woo et al., 2023). Collectively, these observations suggest that microbial enzymatic activity may influence the metabolic fate and cytotoxicity of chemotherapeutic agents through both direct and indirect mechanisms. However, direct evidence linking these specific microbial drug-metabolizing enzymes to treatment outcomes in cervical cancer remains limited, and this represents a critical direction for future investigation.

LPS derived from Gram-negative bacteria prevalent in cervical cancer tissues including *Escherichia coli* and *Prevotella bivia*, has been implicated in therapy resistance as a signaling molecule140. In cervical cancer models, LPS engagement of TLR4 on tumor cells activates MAPK/ERK signaling, upregulates pro-survival factors such as EFNA1 and EDN2, and has been linked to lymph node metastasis. In HPV-positive cervical cancer cell lines, chronic TLR4 activation has been associated with reduced sensitivity to cisplatin-induced apoptosis (Morale et al., 2022). This reduced sensitivity can be attenuated by TLR4 blockade or by interventions that decrease LPS production, pointing to microbial components as modulators of drug response.

**Figure 4:**
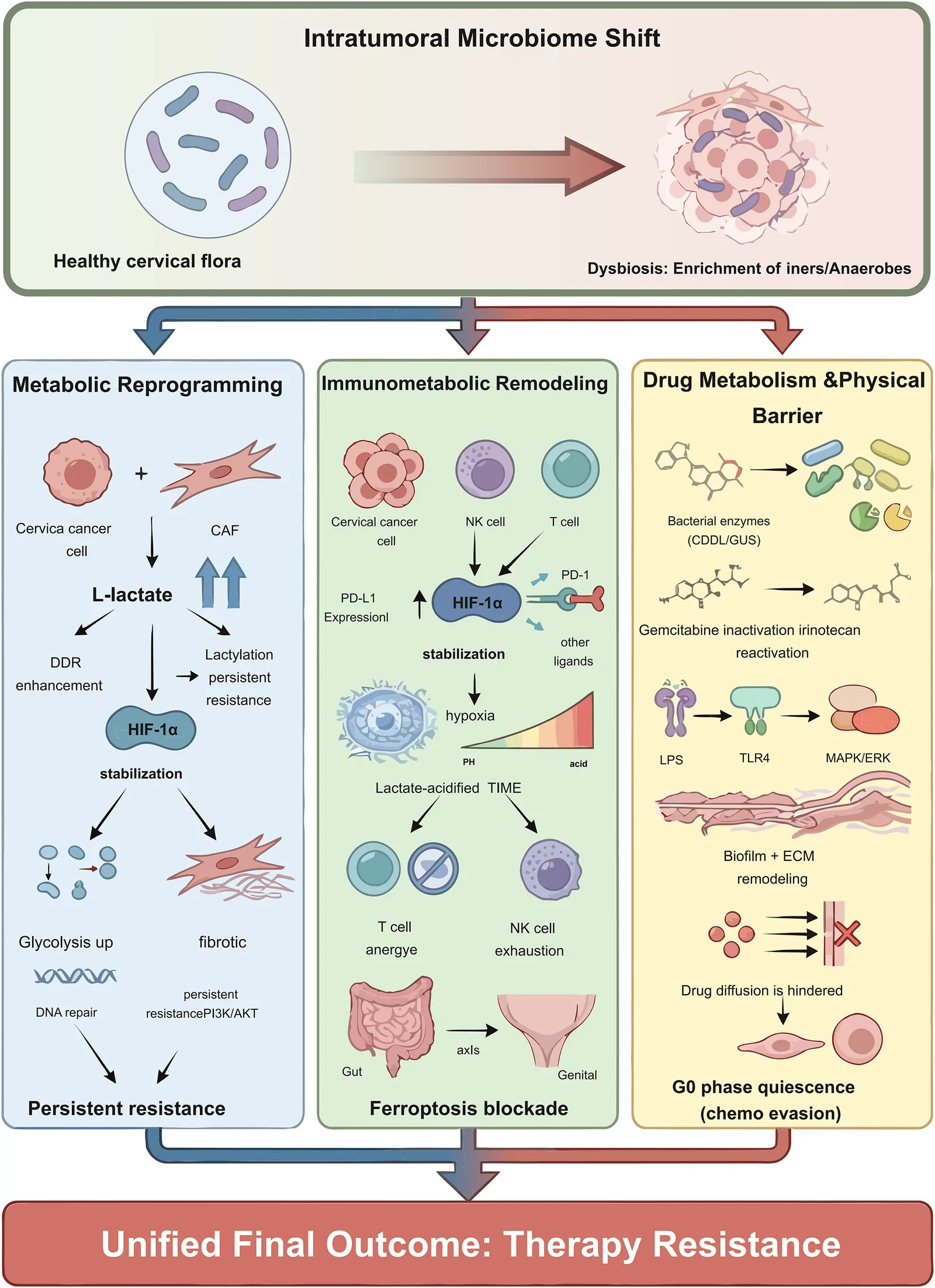
Schematic illustration of therapy resistance mechanisms in cervical cancer. This schematic delineates the key mechanisms driving therapy resistance in cervical cancer. Initiated by a shift in the intratumoral microbiota, featuring enrichment of Lactobacillus iners and a rise in anaerobic bacteria, the process branches into three principal pathways: metabolic reprogramming, immunometabolic remodeling, and drug metabolism alongside barrier formation. These cascades converge to foster resistance to radiotherapy, chemotherapy, and immunotherapy, mediated by HIF-1α stabilization, immunosuppressive microenvironment development, drug modulation, and physical barrier establishment.

In addition to direct biochemical inactivation, microbial communities establish physical barriers that compromise therapeutic efficacy. In HPV-associated cervical lesions, *Gardnerella vaginalis* and other anaerobes frequently form dense biofilms. Biofilm formation by *G. vaginalis* is associated with reduced susceptibility to antibiotics such as metronidazole and clindamycin (Ma et al., 2022). However, direct evidence linking *G. vaginalis* biofilms to cisplatin resistance or impaired drug diffusion specifically in cervical cancer remains limited. Nevertheless, the hypoxic and metabolically dormant interior of the biofilm drives tumor cells into quiescence (G0 phase), thereby rendering them refractory to cycle-active chemotherapies. Separately, *Porphyromonas gingivalis* employs its FimA adhesin to bind host CD151/integrin β1, activating JNK/paxillin signaling. This axis not only promotes metastasis but also upregulates matrix metalloproteinases (MMPs), leading to extracellular matrix (ECM) remodeling. The subsequent elevation in stromal density and interstitial fluid pressure establishes a substantial diffusion barrier, which restricts the penetration of both small-molecule drugs and antibodies into the tumor core. Consequently, residual cancer cells persist within this protected stroma, functioning as reservoirs for recurrence. Clinically, *P. gingivalis*-positive patients exhibit markedly poorer survival, a disparity that is associated with this microbe-driven physical shielding (Huang et al., 2025) (**Figure 4**).

## 5 Conclusions and future perspectives

The oncogenesis and progression of cervical cancer cannot be ascribed exclusively to host genomic mutations; rather, this process can be viewed as an ecological succession shaped by disrupted co-metabolic networks involving both the local and systemic microbiomes. This review systematically delineates the dynamic features of the host–microbiome co-metabolic network across cervical carcinogenesis, emphasizing that as the disease advances, the vaginal microenvironment transitions from a *Lactobacillus crispatus*-dominated state toward a pathological ecosystem enriched with anaerobic pathogens, including *Lactobacillus iners* and *Porphyromonas* species (Dai et al., 2025). This shift is accompanied not only by intensified substrate-level nutrient competition but also by extensive metabolic remodeling at the signaling and translational levels.

While this metabolic reprogramming is closely associated with disease progression, current evidence does not yet support a definitive causal connection to persistent HPV infection or CIN evolution. Certain longitudinal studies indicate that a non-*Lactobacillus*-dominant status, typified by CST IV, predicts persistent high-risk HPV infection and advanced CIN, suggesting that microbial dysbiosis may precede neoplastic lesion formation. Mechanistically, HPV E7 can directly disturb the mucosal barrier by suppressing antimicrobial peptides, thereby restricting the nutritional niche available to lactobacilli (Lebeau et al., 2022). However, the regulatory effects of distinct bacterial species on HPV oncogenes appear notably divergent: *L. crispatus* and *Lactobacillus gasseri* downregulate basal E6/E7 expression in HPV16-transformed cells, correlating with protective phenotypes, whereas *Gardnerella vaginalis* upregulates these oncogenes and is associated with accelerated cell cycle progression (Nicolò et al., 2023). Collectively, these observations point to an intriguing but unconfirmed possibility: the causal relationship between the microbiome and HPV may not be fixed, but rather may shift according to the local microenvironmental context. This context-dependency likely originates from the microenvironment-specific actions of microbial metabolites. For instance, although lactate supports HPV clearance in the healthy vaginal milieu of reproductive-aged women, elevated lactate concentrations within the tumor microenvironment are paradoxically linked to chemoradioresistance via metabolic rewiring. Similarly, SCFAs reinforce epithelial integrity at low concentrations, yet at high concentrations they elicit inflammation and recruit neutrophils (Kang et al., 2023; Zhao Z. et al., 2024). These findings highlight that classifying a microbiota as “beneficial” or “detrimental” requires careful interpretation within specific metabolic and immunological contexts, and future interventions must be precisely titrated regarding both route of administration and dosage.

Although the clinical relevance of microbial metabolites in cervical cancer progression and drug resistance is beginning to gain recognition, translation to clinical practice faces considerable challenges. First, marked inter-individual heterogeneity in co-metabolic profiles arises from variations in geographic location, ethnic dietary habits, endemic diseases, and host genetic backgrounds (Nobels et al., 2025). This heterogeneity affects not only metabolic flux but also the expression and affinity of host receptors such as GPCRs, which may underlie discordant findings across cohorts. Second, during the transition from CIN to invasive carcinoma, the co-metabolic network undergoes phase-specific remodeling of microbial composition and metabolic signatures, accompanied by substantial spatial heterogeneity. Existing cross-sectional study designs, however, are not well suited for capturing these dynamic trajectories, and evidence-based definitions of precise therapeutic windows for distinct pathological stages remain unavailable. These limitations severely constrain efforts to move beyond correlative observations toward validated causation and eventual clinical translation.

Future research should prioritize overcoming these descriptive bottlenecks. In the spatiotemporal domain, well-designed longitudinal cohorts incorporating multi-timepoint sampling and integrating host, microbial, and immune multi-omics data are urgently needed to establish the dynamic causal chains underlying disease progression (Chen et al., 2025). Concurrently, spatial omics technologies should be utilized to uncover the *in situ* mechanistic logic by which pathogenic bacteria contribute to therapeutic resistance. Regarding intervention strategies, given that co-metabolic dysregulation may be involved in refractory disease, clinical validation of vaginal and fecal microbiota transplantation (VMT/FMT) warrants active pursuit. However, a complementary pivot toward synthetic biology-driven precision approaches is also advisable, including the development of engineered LBPs and targeted postbiotics designed to modulate resistance-associated metabolites or block their cognate receptor signaling on demand (Martinelli et al., 2023). Such strategies could ultimately forge new avenues for individualized risk stratification, targeted prophylaxis, and improved therapeutic efficacy.

## Glossary

HR HPV: high risk human papillomavirus
CST: community state type
PCOS: polycystic ovary syndrome
BV: bacterial vaginosis
HIV: human immunodeficiency virus
SCFAs: short-chain fatty acids
IL-1RA: interleukin-1 receptor antagonist
DC: dendritic cell
MHC: major histocompatibility complex
TNF-α: tumor necrosis factor-alpha
TLR: Toll-like receptor
NF-κB: nuclear factor kappa-B
Treg: regulatory T cell
HDAC: histone deacetylase
HDPs: Host defense peptides
HPV E6: human papillomavirus E6 oncoprotein
GLUT1: glucose transporter type 1
ATP: adenosine triphosphate
CIN: cervical intraepithelial neoplasia
CCR6: C-C chemokine receptor type 6
CXCR3: C-X-C chemokine receptor type 3
HGPRT: hypoxanthine-guanine phosphoribosyltransferase
IMP: inosine monophosphate
CC: cervical cancer
SERINC2: Serine Incorporator 2
MMP-8: matrix metalloproteinase-8
EMMPRIN: extracellular matrix metalloproteinase inducer
YAP1: Yes-associated protein 1
HCA1: hydroxycarboxylic acid receptor 1
nCEVs: nano-sized extracellular vesicles
2-HG: 2-hydroxyglutarate
alpha-KG: alpha-ketoglutarate
c-MYC: cellular myelocytomatosis oncogene
HK2: upregulates hexokinase 2
LDHA: lactate dehydrogenase A
lncRNA: long non-coding RNA
NEAT1: nuclear enriched abundant transcript 1
RAC1: Ras-related C3 botulinum toxin substrate 1
MCT-1: monocarboxylate transporter 1
MCT-4: monocarboxylate transporter 4
TCA cycle: tricarboxylic acid cycle
SREBP-1: sterol regulatory element-binding protein 1
FASN: fatty acid synthase
ACCa: acetyl-CoA carboxylase alpha
VEGF-C: vascular endothelial growth factor C
VEGF-D: vascular endothelial growth factor D
ILA: indole-3-lactic acid
AhR: aryl hydrocarbon receptor
ARNT: aryl hydrocarbon receptor nuclear translocator
DREs: dioxin response elements
IDO1: indoleamine 2,3-dioxygenase 1
PAMPs: pathogen-associated molecular patterns
cGAS: cyclic GMP-AMP synthase
STING: stimulator of interferon genes
DNA-PKcs: DNA-dependent protein kinase catalytic subunit
NHEJ: non-homologous end joining
LPS: lipopolysaccharide
STAT3: signal transducer and activator of transcription 3
ISGs: interferon-stimulated genes
ceRNA: competitive endogenous RNA
PD-L1: programmed death-ligand 1
PLC: phospholipase C
PKC: protein kinase C
CAIX: carbonic anhydrase IX
RSAD2: radical S-adenosyl methionine domain containing 2
IFIT1: interferon-induced protein with tetratricopeptide repeats 1
mtROS: mitochondrial reactive oxygen species
NLRP3: NOD-like receptor family pyrin domain containing 3
HIFs: Hypoxia-Inducible Factors**;** DNMT3B, DNA methyltransferase 3 beta
DNMT1: DNA methyltransferase 1
CXCL14: C-X-C motif chemokine ligand 14
MDM2: mouse double minute 2 homolog
FOXO: Forkhead box O
FAO: fatty acid oxidation
CRT: chemoradiation
MCTs: monocarboxylate transporters
HIF-1α: hypoxia-inducible factor 1α
CAFs: cancer-associated fibroblasts
HGF: hepatocyte growth factor
FGF2: fibroblast growth factor 2
DDR: DNA damage repair
ABCB1: ATP-binding cassette subfamily B member 1
NDRG1: myc downstream regulated gene 1
PARP1: poly(ADP-ribose) polymerase 1
PSME3: proteasome activator complex subunit 3
UGCG: UDP glucose ceramide glucosyltransferase
TIME: tumor immune microenvironment
PGE2: prostaglandin E2
MMP: massive matrix metalloproteinase
ECM: extensive extracellular matrix
NK: natural killer
TAMs: tumor associated macrophages
GUS: β-glucuronidase
LBPs: live biotherapeutics
